# Correction to ‘Congenital Lobar Emphysema in an Infant Presenting With Persistent Cough and Progressive Respiratory Symptoms: A Case Report’

**DOI:** 10.1002/ccr3.72677

**Published:** 2026-05-05

F. Abboud, A. A. Muhammad, S. Ali, A. ElHilali, M. A. Amar, and L. Jouria, “Congenital Lobar Emphysema in an Infant Presenting With Persistent Cough and Progressive Respiratory Symptoms: A Case Report,” Clinical Case Reports 14, no. 5 (2026): e72624, https://doi.org/10.1002/ccr3.72624.

In the original publication of this article, there were errors in the author information for Abdeljalil El Hilali and Sawssan Ali.

In the author list and the Author Contribution, the author's name was incorrectly presented as “Abdeljalil ElHilali.” The correct form is “Abdeljalil El Hilali.”

Additionally, two authors' affiliations were incorrectly linked. Abdeljalil El Hilali should be linked to Affiliation 3 (Faculty of Medicine and Pharmacy of Agadir, Ibn Zohr University, Agadir, Morocco) instead of Affiliation 2 and Sawssan Ali should be linked to Affiliation 2 (Children's University Hospital, Damascus, Syria) instead of Affiliation 3.

The online version of this article has been corrected accordingly.

We apologize for this error.

